# Incidence risk of various types of digestive cancers in patients with pre-dialytic chronic kidney disease: A nationwide population-based cohort study

**DOI:** 10.1371/journal.pone.0207756

**Published:** 2018-11-20

**Authors:** Hyung Jung Oh, Hye Ah Lee, Chang Mo Moon, Dong-Ryeol Ryu

**Affiliations:** 1 Ewha Institute of Convergence Medicine, Ewha Womans University Mokdong Hospital, Seoul, Republic of Korea; 2 Research Institute for Human Health Information, Ewha Womans University Mokdong Hospital, Seoul, Republic of Korea; 3 Clinical Trial Center, Ewha Womans University Mokdong Hospital, Seoul, Republic of Korea; 4 Department of Internal Medicine, College of Medicine, Ewha Womans University, Seoul, Republic of Korea; 5 Tissue Injury Defense Research Center, Ewha Womans University, Seoul, Republic of Korea; University of California Irvine, UNITED STATES

## Abstract

Although renal dysfunction is associated with a higher incidence of malignancies, there is no research on the incidence of specific types of digestive cancer in pre-dialytic chronic kidney disease (CKD) patients compared to the general population. This study was conducted on newly diagnosed pre-dialytic CKD patients (n = 35,443) between 2003 and 2013 using the National Health Insurance Service-National Sample Cohort in Korea. The risk of digestive cancer development in pre-dialytic CKD patients was calculated as the standardized incidence ratio (SIR). During a median follow-up of 54.9 months, the risk of digestive cancer in CKD patients was significantly higher than in the cohort population [SIR; 1.54, 95% confidence interval (95% CI); 1.46–1.62], the SIR of pancreatic cancer was 2.21, and the SIRs of hepatoma, colorectal cancer (CRC), bile duct cancer, and gastric cancer were 2.01, 1.60, 1.40, and 1.25, respectively. Moreover, in CKD patients younger than 40 years, the incidence ratios of hepatoma and CRC were remarkably larger compared with the cohort population of the same age (SIR; 5.98 in hepatoma, 4.58 in CRC). However, the incidence of specific types of digestive cancer seemed to be similar, irrespective of sex. In conclusion, digestive cancers were more frequently observed in CKD-diagnosed patients compared with a cohort population in Korea, which suggests that physicians should closely monitor their patients for the incidence of digestive cancer when they are diagnosed with CKD.

## Introduction

Renal function impairment has been shown to be involved in multiple organ diseases [1]. Decreased glomerular filtration rate (GFR) is not only strongly associated with cardiac diseases [2–4] but is also related to non-cardiovascular mortality [5] and a higher incidence of malignancies [6–8]. Moreover, the rate of cancer incidence is higher even in patients with earlier stages of chronic kidney disease (CKD) compared to non-CKD subjects [8–10]. In addition, one recent study demonstrated that an elevated albumin-to-creatinine ratio is significantly associated with increased cancer incidence in a longitudinal population-based study [11]. Furthermore, Wong et al. [12] demonstrated that men, not women, with stage III CKD or worse, had a significantly increased risk of cancer that increased linearly as GFR declined.

Meanwhile, epidemiologic studies have shown that the prevalence of CKD in the United States is over 10% [13, 14] and that the prevalence of CKD stages I-IV increased from 10% in the 1988–1994 period to 13.1% in the 1999–2004 period^14^. In Korea, the total estimated prevalence of CKD for adults ≥ 20 years is 8.2% [15].

Regarding the rates of specific types of cancer in Korea, the incidence of digestive cancer had been increasing until the year 2011, when it reached 176.7/100,000 persons [16]. Although the incidence rate seems to have decreased after 2011 (172.3/100,000 persons in 2013) [17], the incidence rates of gastric cancer and colorectal cancer were still within the top five among all cancers in Korea in 2013. In addition, the developing rate of gastric cancer was particularly high in Korea compared to Caucasian countries and regions [18].

However, to our knowledge, there is no study on the incidence risk of specific types of digestive cancer in patients with CKD compared to the cohort population. The standardized incidence ratio (SIR) represents a relative excess or decrement in the actual event experience of the study group in relation to what was expected if the incident experience was equal in the reference population. In Korea, the National Health Insurance Service (NHIS) has released a nationally representative cohort database (2.2% of the Korean population in 2002) based on demographics, medical treatment, procedures, and disease diagnoses. Therefore, this study was conducted on newly diagnosed patients with pre-dialytic CKD (n = 35,443) between 2003 and 2013 using the NHIS-National Sample Cohort (NHIS-NSC) in Korea. We investigated the risk of digestive cancer development in patients with pre-dialytic CKD compared with the cohort population using the SIR.

## Results

### Baseline characteristics

Of 898,140 individuals in the cohort populations, 510,649 individuals (56.9%) were younger than 40 years, and 446,231 (49.7%) were men (Table 1). Moreover, 19,683 individuals were diagnosed with cancer in the cohort population during the follow-up periods (data not shown). In the 35,443 pre-dialytic CKD patients, most of these patients were in the age range of 40 to 64 (57.7%). There were 17,147 (48.4%) male patients, and income levels in the enrolled participants are presented in Table 1. When these patients were divided into two groups based on the development of digestive cancer, 1,443 (4.1%) patients were diagnosed with digestive cancer during the follow-up period. As seen in Table 1, there was a higher incidence of digestive cancer in the older age group. The digestive cancer group had more male patients (57.2%) than the digestive cancer-free group (48.0%) (*p* < 0.0001), but there was no significant difference in income levels between the two groups (Table 1).

**Table 1:** Baseline characteristics in patients with pre-dialytic CKD.

		Cohort population N = 898,140	Pre-dialytic CKD		
			Without digestive cancer N = 34,000 (95.9%)	With digestive cancer N = 1,443 (4.1%)	*p*-value
**Age, years**					< 0.0001
	**< 40**	510,649 (56.9%)	6,720 (19.8%)	57 (4.0%)	
	**40 to 64**	307,991 (34.3%)	19,681 (57.9%)	755 (52.3%)	
	**≥65 years**	79,500 (8.8%)	7,599 (22.4%)	631 (43.7%)	
**Male, n (%)**		446,231 (49.7%)	16,322 (48.0%)	825 (57.2%)	< 0.0001
Income level*					0.806
	**≤ 20**^**th**^	144,035 (16.0%)	5,869 (17.3%)	258 (17.9%)	
	**21 to 40**^**th**^	140,574 (15.7%)	4,743 (14.0%)	201 (13.9%)	
	**41 to 60**^**th**^	171,690 (19.1%)	5,740 (16.9%)	238 (16.5%)	
	**61 to 80**^**th**^	202,662 (22.6%)	7,403 (21.8%)	298 (20.7%)	
	**≥ 81**^**th**^	239,179 (26.6%)	10,245 (30.1%)	448 (31.1%)	

Data are expressed as n (%)

Abbreviations; CKD, chronic kidney disease

*Income level; Income class is divided into five categories by Korean National Health Insurance. Medical aid beneficiaries are categorized as below 20^th^ percentile, and the others are included in the other classes according to their income levels.

### Standardized incidence ratio of digestive cancer in pre-dialytic CKD patients compared with the cohort population

During the median follow-up period of 54.9 (interquartile range; 24.0–93.3) months, an increased overall digestive cancer risk in patients with CKD was observed compared with the cohort population [SIR; 1.54, 95% confidence interval (CI); 1.46–1.62]. Moreover, the SIRs of colorectal, gastric, hepatoma, pancreatic, and bile duct cancers in CKD patients were 1.60, 1.25, 2.01, 2.21, and 1.40, respectively. However, there were no significant differences in incidence ratio for gall bladder, esophageal, and small bowel cancers between the CKD-diagnosed population and the cohort population (Table 2).

**Table 2:** Standardized incidence ratio of digestive cancer in pre-dialytic CKD patients compared with cohort population.

Cancer locations	No. observed	No. expected	SIR	95% CI
**Digestive cancer**	1443	937.89	1.54	1.46–1.62
**Colorectal cancer**	487	305.01	1.60	1.45–1.74
**Gastric cancer**	382	306.46	1.25	1.12–1.37
**Hepatoma**	240	119.33	2.01	1.76–2.27
**Pancreatic cancer**	171	77.25	2.21	1.88–2.55
**Bile duct cancer**	95	67.75	1.40	1.12–1.68
**Gall bladder cancer**	39	28.31	1.38	0.95–1.81
**Esophageal cancer**	23	26.16	0.88	0.52–1.24
**Small bowel cancer**	6	7.63	0.79	0.16–1.42

Abbreviations; CKD, chronic kidney disease; No, number; SIR, standardized incidence ratio; CI, confidence interval

**Fig 1:**
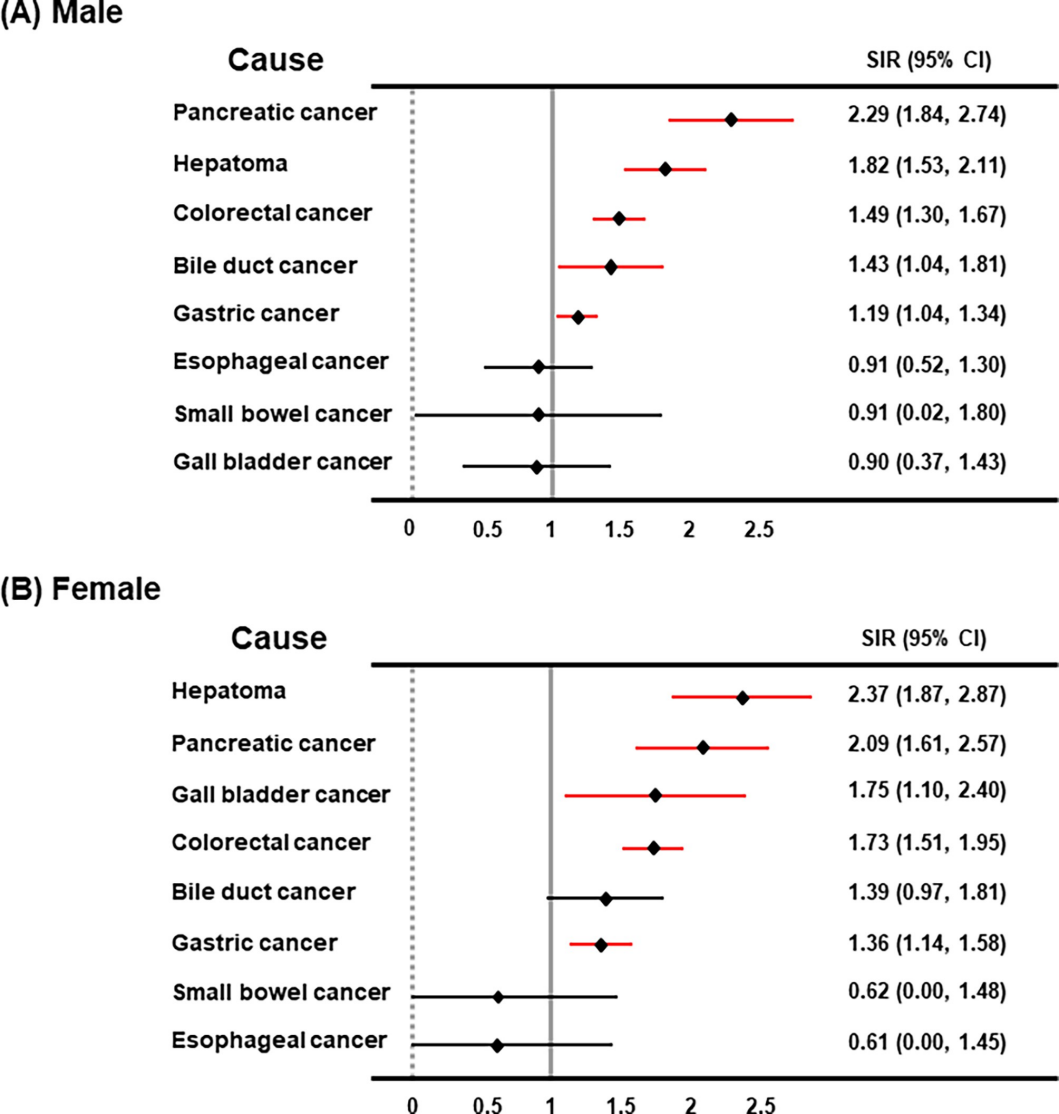
Standardized incidence ratio of digestive cancer in pre-dialytic CKD patients compared with the cohort population (men and women). Abbreviations: CKD, chronic kidney disease; No, number; SIR, standardized incidence rate; CI, confidence interval.

Next, we investigated the relative incidence risk of digestive cancer between men and women. The overall incidence ratios of digestive cancer in men and women were higher in CKD-diagnosed patients compared with those in the cohort population (Table 3). Additionally, pancreatic cancer, hepatoma, colorectal cancer, and gastric cancer were revealed to be more frequently observed in CKD-diagnosed patients compared to those in the cohort population, irrespective of gender. However, bile duct cancer was more observed in male CKD patients compared with in cohort population, while gall bladder cancer was more common in female CKD patients compared to in cohort population (Fig 1).

**Table 3:** Standardized incidence ratio of overall digestive cancer in CKD diagnosed patients compared with cohort population stratified by gender and age.

Digestive cancer		No. observed	No. expected	SIR	95% CI
**Gender**					
	Male	825	571.33	1.44	1.35–1.54
	Female	618	370.26	1.67	1.54–1.80
**Age, years**					
	< 40	20	6.16	3.25	1.82–4.67
	40 to 64	457	297.15	1.54	1.40–1.68
	≥ 65	966	634.58	1.52	1.43–1.62

Abbreviations; CKD, chronic kidney disease; No, number; SIR, standardized incidence rate; CI, confidence interval

**Fig 2:**
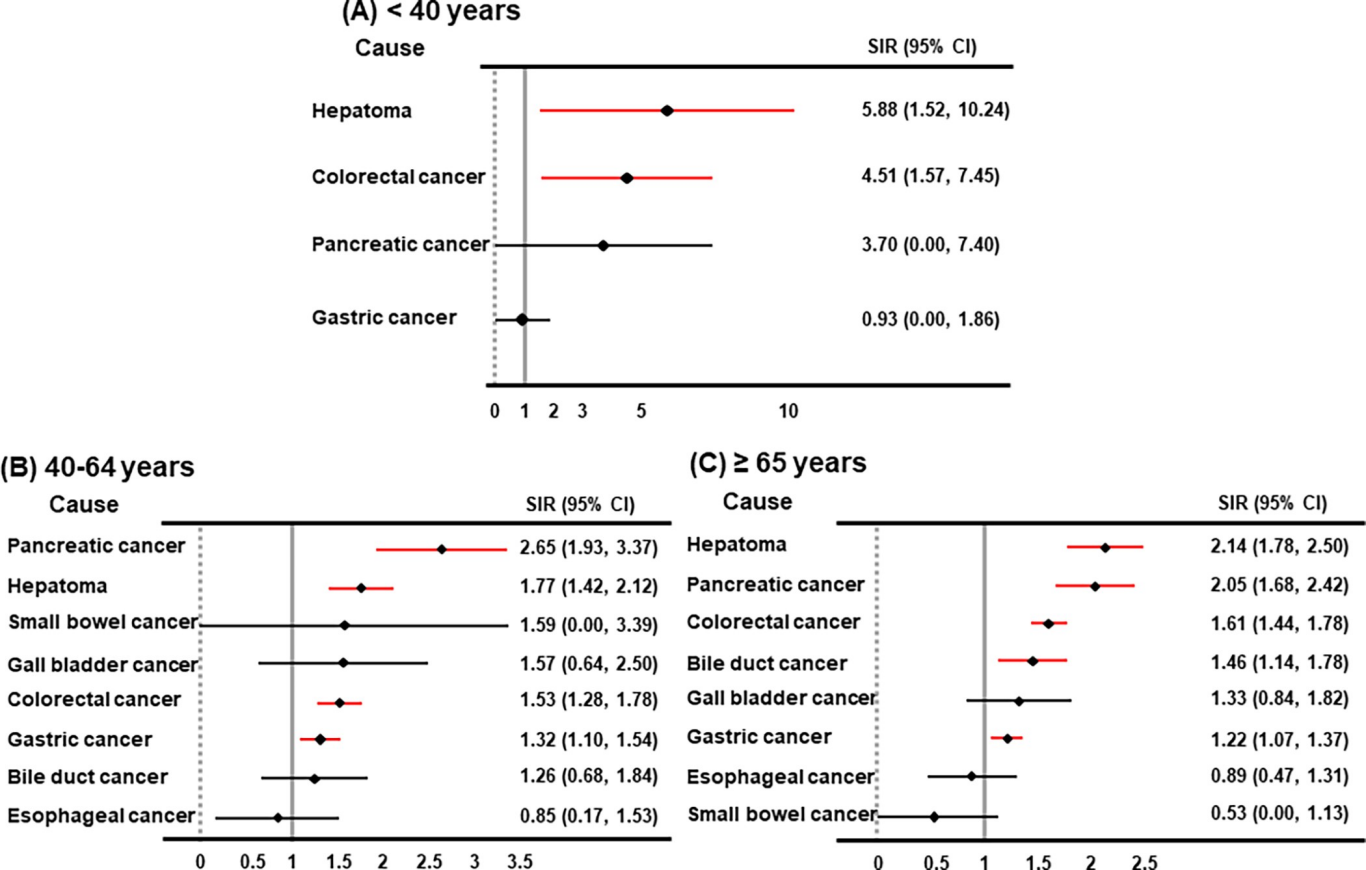
Standardized incidence ratio of digestive cancer in pre-dialytic CKD patients compared with the cohort population, stratified by age (under 40 years, 40 to 64 years, and ≥65 years). Abbreviations: CKD, chronic kidney disease; No, number; SIR, standardized incidence ratio; CI, confidence interval.

When we stratified these patients into three groups based on age (< 40 years, 40 to 64 years, and ≥ 65 years), the SIR for digestive cancer was 3.25 in the patients less than 40 years, 1.54 in the patients between 40 to 64 years, and 1.52 in the patients 65 years and older (Table 3). Moreover, Fig 2 showed that hepatoma (SIR, 2.14), pancreatic cancer (SIR, 2.05), colorectal cancer (SIR, 1.61), bile duct cancer (SIR, 1.46), and gastric cancer (SIR, 1.22) were more frequently observed in CKD patients of 65 years and older compared with in cohort population with the same age (≥65 years). Furthermore, in the CKD patients with 40–64 years, pancreatic cancer (SIR, 2.66), hepatoma (SIR, 1.78), colorectal cancer (SIR, 1.51), and gastric cancer (SIR, 1.30) were more common compared to the cohort population with the same age (40 to 64 years). In the patients less than 40 years old, hepatoma (SIR, 5.98) and colorectal cancer (SIR, 4.58) were more observed in CKD patients compared with the cohort population with the same age (<40 years) (Fig 2).

When the CKD subjects were divided into two groups (early detection vs. late detection), based on the follow-up duration from CKD diagnosis (1-year), the observed number of digestive cancer cases in the early- and late-detection groups was 483 (33.5%) and 960 (66.5%), respectively (S1 Table). In addition, S1 Table showed that all the SIRs were decreased in the late-detection group compared with those in all the enrolled patients (Table 2). Moreover, the statistical significance of the SIRs in gastric cancer and bile duct cancer disappeared in the late detection group. These findings indicate that digestive cancer was relatively more common in the early-detection group.

## Discussion

The results of our study showed that the incidence risk of digestive cancer in CKD patients was significantly higher than that in the cohort population. In addition, the SIRs of hepatoma and colorectal cancers were relatively higher in CKD patients less than 40 years compared with those patients 40 years and over. However, the incidence risk for specific types of digestive cancer seemed to be similar irrespective of sex. To date, there are many studies on the association between decreased renal function and cancer incidence [6–12]. However, there is no study on the incidence risk of specific types of digestive cancer in patients with CKD compared with the general population.

Although the precise biological reason why digestive cancer risk was higher in CKD patients compared to the general population cannot be clearly explained, previous research has indicated that it may be associated with vitamin D deficiency [19, 20], uremic factors [12], or CKD itself as a proinflammatory state [21–23]. However, as seen in S1 Table, all the SIRs were decreased in the late-detection group compared with those in the enrolled population (Table 2), and no significant SIRs of gastric cancer and bile duct cancer were observed in the late-detection group, indicating that the incidence of digestive cancer was relatively more common in the early-detection group. Taken together, we deduce that digestive cancer might be observed incidentally by diagnostic tools during the work-up for CKD, even though there is little relation between CKD diagnosis and the occurrence of digestive cancer. Moreover, the reason for the higher incidence ratio for gastric and colorectal cancers in this study may be partially explained by the fact that screening examinations for these cancers are more commonly performed in Korea. In the National Cancer Screening Program, the Korean government provides gastric cancer screening, including a biennial upper endoscopy or upper gastrointestinal series, for individuals ≥40 years of age [24] and colorectal cancer screening, including an annual fecal occult blood test (FOBT), for those ≥50 years of age [25]. Therefore, we presumed that the SIRs for these cancers might be relatively lower in CKD patients when comparing hepatoma and pancreatic cancers.

In contrast, radiation contrast agent for computerized tomography (CT) is not generally recommended when patients are suffering from CKD [26–29] to avoid the occurrence of contrast-induced nephropathy. Additionally, contrast imaging is not a routine examination at health check-ups in Korea. Instead, a kidney ultrasonography is usually chosen to identify kidney shape, size, and echogenicity when patients are diagnosed with CKD [28, 29]. We can deduce that it is possible that such ultrasonography may unexpectedly provide more information on hidden digestive cancers (e.g., hepatoma or pancreatic cancers) to physicians. This means that the SIRs of hepatoma and pancreatic cancers could be higher than those of gastric and colorectal cancers. Moreover, the incidence of hepatoma in CKD patients may be increased due to viral hepatitis B and C. We investigated comorbidities such as hepatitis B or C in the whole sample cohort compared to the CKD-diagnosed patients (S2 Table), and the prevalence of hepatitis B or C was similar between the two groups. When we compared the incidence ratio of hepatoma according to the presence of the comorbidity of hepatitis B or C (S3 and S4 Tables), hepatoma was more frequently observed in the subjects with hepatitis B or C compared with those without hepatitis B or C in both the CKD-diagnosed patients and the whole cohort population, as we expected.

Based on these data, the incidence risk for specific types of digestive cancer was not different between men and women. However, the SIRs for hepatoma and colorectal cancers were greater in CKD patients younger than 40 years than those in CKD patients 40 years or over. It is difficult to explain the reason why they were higher in this age group, but it may be because the observed number was significantly smaller in this age group than the other age groups (Fig 2). Therefore, the data from this study should be interpreted with caution. The data suggest that physicians may pay closer attention to their younger-aged CKD patients with respect to an increase in the incidence risk of hepatoma and colorectal cancers compared with the remaining cohort population of the same age.

There were several limitations to our study. First, the NHIS-NSC was established based on administrative data from health insurance claims instead of clinical data that includes disease progress. Therefore, we were unable to investigate patients based on stratified CKD stage. Second, the diagnosis of CKD and digestive cancer was based on a number of diagnostic codes, and it was not easy to verify the diagnoses from the codes. However, Kim [30] and Yoo et al. [31] defined CKD using the 10^th^ revision of the international classification of diseases (ICD-10) code in their recently published papers, and we defined CKD using the same method in this study. In addition, the diagnostic codes for digestive cancer were also used in previously published manuscripts [32, 33]. Although we were limited in our definition of diagnosis by using diagnostic codes, we presume that the codes could accurately designate CKD or digestive cancer in the current study. Third, this study did not evaluate some cancer-related risk factors, such as smoking and alcohol consumption, exercise and dietary habits because these data were unavailable in our cohort. These confounding factors may affect the incidence of digestive cancer. However, in previous epidemiological studies [10, 34], SIR was used to assess the health risks of the population of interest. Further study, including all possible confounding factors will be needed. Additionally, uncertainty regarding misclassification of disease might result in attenuated results, but several specific types of cancer showed significant results. To reduce that bias, we defined disease based on methods used in national reports and statistics. In the present study, we used the incidence rate of the cohort population to estimate the SIR. The bias introduced by data collection systems should be reduced as a result. However, the findings should be interpreted with caution because this study was conducted in patients with CKD that was only defined by diagnostic code. Fourth, the study used a retrospective design and selection bias could not be completely avoided. Fifth, in this study, because we have focused on the incidence of digestive cancer in patients with pre-dialytic CKD, we have excluded those with end-stage renal disease (ESRD) or kidney transplantation (KT) before diagnosis of digestive cancer. We also recognize the possibility of a selection bias by excluding the transferring patients from CKD to ESRD or KT. However, the effect of pre-dialytic CKD on digestive cancer incidence would have been difficult to study if we had included patients with ESRD or those who underwent KT. Despite these limitations, this study presented relative cancer incidence risk, focusing especially on digestive cancer, in CKD patients compared with the cohort population using SIRs. Our results were obtained from a national representative sample using NHIS-NSC data. In addition, the estimated SIRs and rankings based on the NCR, which is representative of Korean cancer incidence risk, were similar to findings from the cohort population (S5 Table) and support the validity of our findings.

In conclusion, digestive cancers were more frequently observed in CKD-diagnosed patients compared with a cohort population in Korea. In particular, hepatoma and colorectal cancer were more frequently observed in CKD-diagnosed patients less than 40 years compared with the CKD diagnosed patients 40 years and over, indicating that physicians likely pay closer attention to the increased incidence risk of digestive cancer when their patients are suffering from CKD.

## Subjects and methods

### Data resource

The investigation was performed using data from the NHIS-NSC in the Republic of Korea. The cohort was composed of 2.2% of the total eligible Korean population (baseline population = 1,025,340 people), which was selected as a representative sample in 2002 using systematic stratified random sampling. The data contained information for demographics such as age groups (<1 years, 1–4 years, 5-year age groups between 5 and84 years, and ≥85 years), sex, income levels, healthcare utilization, prescriptions, and diagnostic codes based on the ICD-10. Information related to deaths was also provided from the Korea National Statistical Office, with follow-up data from 2002 to 2013 available. Detailed information about the NHIS-NSC can be found in a previously published paper [35]. This study was approved by the Institutional Review Board of the Ewha Womans University Mokdong Hospital (EUMC 2015-05-049). The need for informed consent from patients was waived due to the retrospective design of the study.

### Study sample

**Fig 3:**
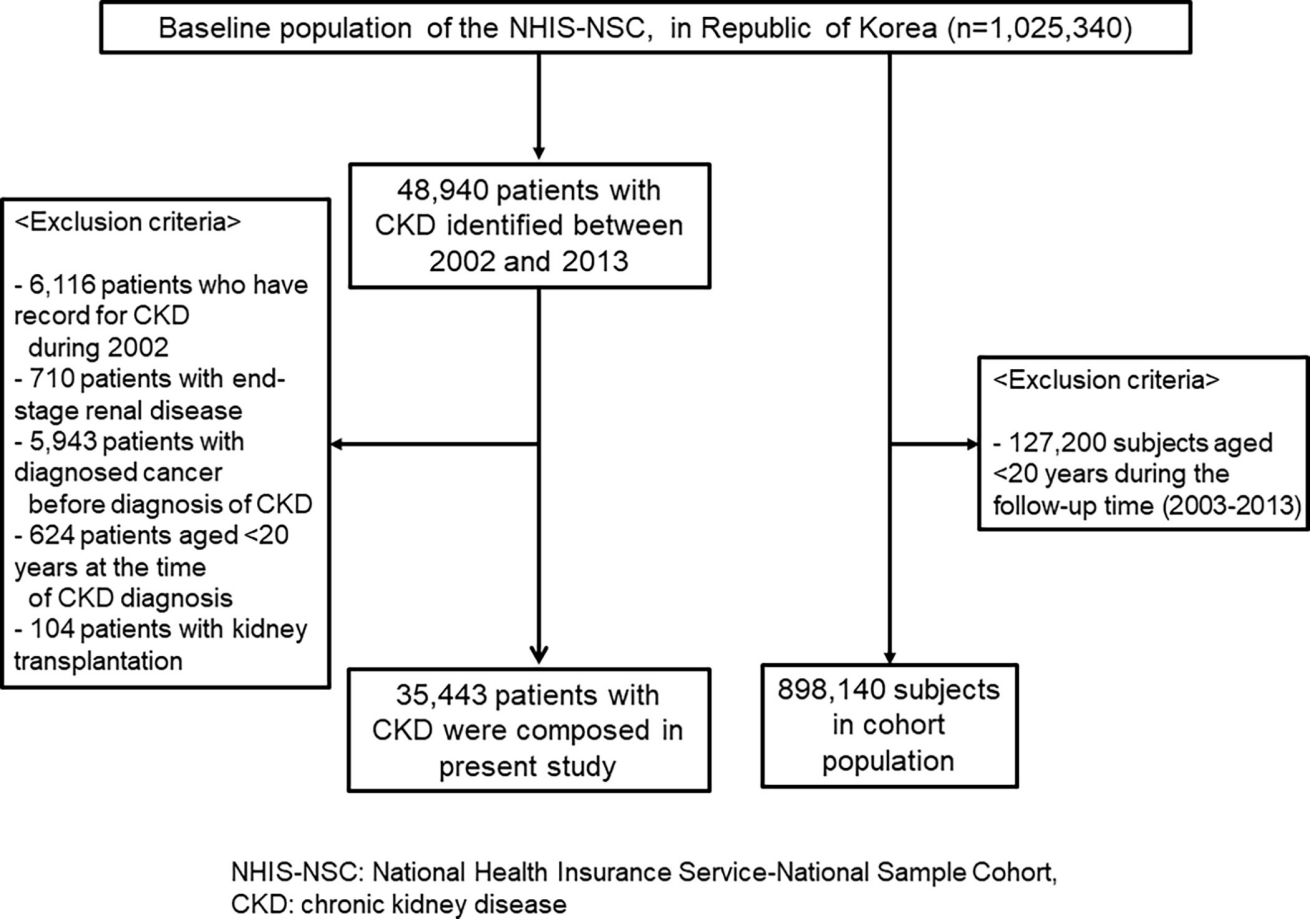
Study flowchart. Abbreviations: NHIS-NSC: National Health Insurance Service-National Sample Cohort; CKD, chronic kidney disease.

We defined pre-dialytic CKD as follows: those who have insurance claims with the diagnostic codes of 'N18' (chronic kidney disease), 'N19' (unspecified kidney failure), 'I12' (hypertensive renal disease), 'I13' (hypertensive heart and renal disease), 'E10.2' (Type 1 diabetes mellitus with renal complications), 'E11.2' (Type 2 diabetes mellitus with renal complications), 'E13.2' (other specified diabetes mellitus with renal complications), and 'E14.2' (unspecified diabetes mellitus with renal complications) from 1 January 2002 to 31 Dec**e**mber 2013 (n = 48,940). However, those who were diagnosed with CKD in 2002 were not included in the current study, because the information about their disease history was inaccurate (n = 6,116). Moreover, we excluded cases who had already had cancer or been diagnosed with a malignancy before 2003 and investigated the patients who were diagnosed with new digestive cancers during the years 2003 to 2013. Of those patients, anyone diagnosed with ESRD (n = 710) before diagnosis of digestive cancer was also excluded from the study. ESRD was identified when subjects had insurance claims with a regular dialysis treatment code for 90 ± 7 days after the date of the first dialysis. Additionally, subjects diagnosed with any cancer before the diagnosis of CKD (n = 5,943) and who were under 20 years of age at the time of CKD diagnosis (n = 624), and 104 patients who underwent KT before diagnosis of digestive cancer were excluded. Finally, a total of 35,443 patients were included in the present study (Fig 3).

### Identification of digestive cancer

To apply a precise definition of digestive cancer, we defined it in detail as follows: esophageal cancer (‘C15’), gastric cancer (‘C16’), small bowel cancer (‘C17’), colorectal cancer (‘C18-20’), hepatoma (‘C22.0’), bile duct cancer (‘C22.1’ and ‘C24’), gall bladder cancer (‘C23’), and pancreatic cancer (‘C25’).

In general, the National Cancer Registry (NCR) reports on the annual incidence rate of 61 types of cancer but does not report sub-categorization with decimal places. For that reason, estimations of the SIRs according to the detailed definition of the cancer using NCR data was limited. Therefore, to calculate the SIRs, we investigated the cancer rates of the complete NHIS-NSC data (cohort population) for adults aged 20 years and older from 2003 to 2013, which were considered as reference cancer rates and compared them with those in patients with CKD. Because the information on risk factors in all cohort subjects was incomplete, we could not use Cox and Poisson regression. In addition, an indirect method, such as SIR, is considered particularly useful either when stratum-specific risks or rates are missing in one of the groups under comparison or when the study group(s) is (are) small [36]. Thus, we estimated the SIR based on the available information. To assess the validity of this method, we compared SIRs of digestive cancer among the cohort population to national data from the NCR. Compared with the incidence rate in the NCR according to the definition of cancer incidence, the incidence rate of the cohort population, which is defined as hospitalization due to digestive cancer, was close to 1.0, whereas the incidence rate, which was defined as the case of one or more claims regardless of inpatient or outpatient settings, was 2.6 times higher (S5 Table). Accordingly, digestive cancer incidence was defined as first admission with the diagnostic code of a digestive cancer between 1 January 2003 and 31 December 2013. In addition, previous Korean studies which were conducted using insurance claims data have also applied the same criteria for cancer incident cases [32, 33].

### Cancer risk and statistical analysis

We estimated SIRs with 95% CIs to assess the incidence risk of digestive cancer among patients with pre-dialytic CKD. SIRs were calculated as the observed number of cancer cases divided by the expected number of cases. The expected number of cancer cases was estimated as the age- (5-year intervals) and calendar year-specific incidence rates of the cohort population multiplied by the number of persons at risk.

Moreover, SIRs were estimated after stratification by age group (<40 years, 40–64 years, and 65+ years) or sex at baseline. We also estimated SIRs with 95% CIs for the subtypes of digestive cancer, and all the statistical analyses were carried out using SAS version 9.4 (SAS Institute, Cary, NC, USA). A *p*-value <0.05 in two-sided tests was considered statistically significant.

## Supporting information

S1 Table Standardized incidence ratio of digestive cancer in pre-dialytic CKD patients compared with cohort population in late detection group. (DOC)

S2 Table The presence of comorbidities, hepatitis B or C in whole sample cohort and CKD-diagnosed patients. (DOC)

S3 Table The incidence of hepatoma according to the presence of hepatitis B in CKD-diagnosed patients and whole sample cohort. (DOC)

S4 Table The incidence of hepatoma according to the presence of hepatitis C in CKD-diagnosed patients and whole sample cohort. (DOC)

S5 Table Comparison of incidence of digestive cancers according to disease definition in data from the National Health Insurance Service-National Sample Cohort with National Cancer Registry data in Korea for 2003. (DOC)
