# Supplementary material for: Incidence risk of various types of digestive cancers in patients with pre-dialytic chronic kidney disease: A nationwide population-based cohort study
Source: PLoS One. 2018 Nov 20;13(11):e0207756. doi: 10.1371/journal.pone.0207756 (PMC6245741; doi:10.1371/journal.pone.0207756)
Supplement: S1 Table — (DOC) [file pone.0207756.s001.doc]

**S1 Table. Standardized incidence ratio of digestive cancer in pre-dialytic CKD patients compared with cohort** population in late detection group*

|  | **Late detection group** | | | |
| --- | --- | --- | --- | --- |
|  | **No. observed** | **No. expected** | **SIR** | **95% CI** |
| **Digestive cancer** | 960 | 899.30 | 1.07 | 1.00-1.14 |
| **Colorectal cancer** | 338 | 292.71 | 1.15 | 1.03-1.28 |
| **Gastric cancer** | 280 | 296.75 | 0.94 | 0.83-1.05 |
| **Hepatoma** | 151 | 113.60 | 1.33 | 1.12-1.54 |
| **Pancreatic cancer** | 90 | 71.73 | 1.25 | 1.00-1.51 |
| **Bile duct cancer** | 54 | 64.44 | 0.84 | 0.61-1.06 |
| **Gall bladder cancer** | 28 | 27.31 | 1.03 | 0.65-1.41 |
| **Esophageal cancer** | 15 | 25.32 | 0.59 | 0.29-0.89 |
| **Small bowel cancer** | 4 | 7.44 | 0.54 | 0.01-1.06 |

Abbreviations; CKD, chronic kidney disease; No, number; SIR, standardized incidence rate; CI, confidence interval

*late detection group; the group in which digestive cancer was observed over 1 year from CKD diagnosis

. The presence of comorbidities, hepatitis B or C in Whole Sample Cohort and CKD-diagnosed patients

|  |  |  |
| --- | --- | --- |
|  |  |  |
|  |  |  |

. The incidence of hepatoma according to the presence of hepatitis B in CKD-diagnosed patients and whole Sample Cohort

|  |  | | |  | | |
| --- | --- | --- | --- | --- | --- | --- |
|  |  | |  |  | |  |
|  |  |  |  |  |  |  |
|  |  |  |  |  |  |  |
|  |  |  |  |  |  |  |
|  |  |  |  |  |  |  |

. The incidence of hepatoma according to the presence of hepatitis C in CKD-diagnosed patients and whole Sample Cohort

|  |  | | |  | | |
| --- | --- | --- | --- | --- | --- | --- |
|  |  | |  |  | |  |
|  |  |  |  |  |  |  |
|  |  |  |  |  |  |  |
|  |  |  |  |  |  |  |
|  |  |  |  |  |  |  |

. Comparison of incidence of digestive cancers according to disease definition in data from the National Health Insurance Service-National Sample Cohort with National Cancer Registry data in Korea for 2003

|  |  | | |  |  |  |
| --- | --- | --- | --- | --- | --- | --- |
|  |  |  |
|  |  |  |  |  |  |  |
|  |  |  |  |  |  |  |
|  |  |  |  |  |  |  |
|  |  |  |  |  |  |  |
|  |  |  |  |  |  |  |
|  |  |  |  |  |  |  |
|  |  |  |  |  |  |  |
|  |  |  |  |  |  |  |
|  |  |  |  |  |  |  |
|  |  |  |  |  |  |  |
|  |  |  |  |  |  |  |
|  |  |  |  |  |  |  |
|  |  |  |  |  |  |  |
|  |  |  |  |  |  |  |
|  |  |  |  |  |  |  |
