# Supplementary material for: Incidence risk of various types of digestive cancers in patients with pre-dialytic chronic kidney disease: A nationwide population-based cohort study
Source: PLoS One. 2018 Nov 20;13(11):e0207756. doi: 10.1371/journal.pone.0207756 (PMC6245741; doi:10.1371/journal.pone.0207756)
Supplement: S2 Table — (DOC) [file pone.0207756.s002.doc]

population in late detection group*

|  |  | | | |
| --- | --- | --- | --- | --- |
|  |  |  |  |
|  |  |  |  |  |
|  |  |  |  |  |
|  |  |  |  |  |
|  |  |  |  |  |
|  |  |  |  |  |
|  |  |  |  |  |
|  |  |  |  |  |
|  |  |  |  |  |
|  |  |  |  |  |

**S2 Table**. The presence of comorbidities, hepatitis B or C in Whole Sample Cohort and CKD-diagnosed patients

|  | **Hepatitis B** | **Hepatitis C** |
| --- | --- | --- |
| **Whole Sample Cohort** | 2.65% | 0.92% |
| **CKD-diagnosed patients** | 3.05% | 1.03% |

Abbreviations; CKD, chronic kidney disease

. The incidence of hepatoma according to the presence of hepatitis B in CKD-diagnosed patients and whole Sample Cohort

|  |  | | |  | | |
| --- | --- | --- | --- | --- | --- | --- |
|  |  | |  |  | |  |
|  |  |  |  |  |  |  |
|  |  |  |  |  |  |  |
|  |  |  |  |  |  |  |
|  |  |  |  |  |  |  |

. The incidence of hepatoma according to the presence of hepatitis C in CKD-diagnosed patients and whole Sample Cohort

|  |  | | |  | | |
| --- | --- | --- | --- | --- | --- | --- |
|  |  | |  |  | |  |
|  |  |  |  |  |  |  |
|  |  |  |  |  |  |  |
|  |  |  |  |  |  |  |
|  |  |  |  |  |  |  |

. Comparison of incidence of digestive cancers according to disease definition in data from the National Health Insurance Service-National Sample Cohort with National Cancer Registry data in Korea for 2003

|  |  | | |  |  |  |
| --- | --- | --- | --- | --- | --- | --- |
|  |  |  |
|  |  |  |  |  |  |  |
|  |  |  |  |  |  |  |
|  |  |  |  |  |  |  |
|  |  |  |  |  |  |  |
|  |  |  |  |  |  |  |
|  |  |  |  |  |  |  |
|  |  |  |  |  |  |  |
|  |  |  |  |  |  |  |
|  |  |  |  |  |  |  |
|  |  |  |  |  |  |  |
|  |  |  |  |  |  |  |
|  |  |  |  |  |  |  |
|  |  |  |  |  |  |  |
|  |  |  |  |  |  |  |
|  |  |  |  |  |  |  |
