# Supplementary material for: Incidence risk of various types of digestive cancers in patients with pre-dialytic chronic kidney disease: A nationwide population-based cohort study
Source: PLoS One. 2018 Nov 20;13(11):e0207756. doi: 10.1371/journal.pone.0207756 (PMC6245741; doi:10.1371/journal.pone.0207756)
Supplement: S4 Table — (DOC) [file pone.0207756.s004.doc]

population in late detection group*

|  |  | | | |
| --- | --- | --- | --- | --- |
|  |  |  |  |
|  |  |  |  |  |
|  |  |  |  |  |
|  |  |  |  |  |
|  |  |  |  |  |
|  |  |  |  |  |
|  |  |  |  |  |
|  |  |  |  |  |
|  |  |  |  |  |
|  |  |  |  |  |

. The presence of comorbidities, hepatitis B or C in Whole Sample Cohort and CKD-diagnosed patients

|  |  |  |
| --- | --- | --- |
|  |  |  |
|  |  |  |

. The incidence of hepatoma according to the presence of hepatitis B in CKD-diagnosed patients and whole Sample Cohort

|  |  | | |  | | |
| --- | --- | --- | --- | --- | --- | --- |
|  |  | |  |  | |  |
|  |  |  |  |  |  |  |
|  |  |  |  |  |  |  |
|  |  |  |  |  |  |  |
|  |  |  |  |  |  |  |

**S4 Table**. The incidence of hepatoma according to the presence of hepatitis C in CKD-diagnosed patients and whole Sample Cohort

|  | CKD-diagnosed patients | | | Whole Sample Cohort | | |
| --- | --- | --- | --- | --- | --- | --- |
|  | Hepatoma | | Total | Hepatoma | | Total |
| Hepatitis C | No | Yes |  | No | Yes |  |
| No | 34,856 | 223 (0.6%) | 35,079 | 992,128 | 2,493 (0.3%) | 994,621 |
| Yes | 347 | 17 (4.7%) | 364 | 7,590 | 477 (5.9%) | 8,067 |
| Total | 35,203 | 240 | 35,443 | 999,718 | 2,970 | 1,002,688 |

Abbreviations; CKD, chronic kidney disease

. Comparison of incidence of digestive cancers according to disease definition in data from the National Health Insurance Service-National Sample Cohort with National Cancer Registry data in Korea for 2003

|  |  | | |  |  |  |
| --- | --- | --- | --- | --- | --- | --- |
|  |  |  |
|  |  |  |  |  |  |  |
|  |  |  |  |  |  |  |
|  |  |  |  |  |  |  |
|  |  |  |  |  |  |  |
|  |  |  |  |  |  |  |
|  |  |  |  |  |  |  |
|  |  |  |  |  |  |  |
|  |  |  |  |  |  |  |
|  |  |  |  |  |  |  |
|  |  |  |  |  |  |  |
|  |  |  |  |  |  |  |
|  |  |  |  |  |  |  |
|  |  |  |  |  |  |  |
|  |  |  |  |  |  |  |
|  |  |  |  |  |  |  |
