# Supplementary material for: Incidence risk of various types of digestive cancers in patients with pre-dialytic chronic kidney disease: A nationwide population-based cohort study
Source: PLoS One. 2018 Nov 20;13(11):e0207756. doi: 10.1371/journal.pone.0207756 (PMC6245741; doi:10.1371/journal.pone.0207756)
Supplement: S5 Table — (DOC) [file pone.0207756.s005.doc]

population in late detection group*

|  |  | | | |
| --- | --- | --- | --- | --- |
|  |  |  |  |
|  |  |  |  |  |
|  |  |  |  |  |
|  |  |  |  |  |
|  |  |  |  |  |
|  |  |  |  |  |
|  |  |  |  |  |
|  |  |  |  |  |
|  |  |  |  |  |
|  |  |  |  |  |

. The presence of comorbidities, hepatitis B or C in Whole Sample Cohort and CKD-diagnosed patients

|  |  |  |
| --- | --- | --- |
|  |  |  |
|  |  |  |

. The incidence of hepatoma according to the presence of hepatitis B in CKD-diagnosed patients and whole Sample Cohort

|  |  | | |  | | |
| --- | --- | --- | --- | --- | --- | --- |
|  |  | |  |  | |  |
|  |  |  |  |  |  |  |
|  |  |  |  |  |  |  |
|  |  |  |  |  |  |  |
|  |  |  |  |  |  |  |

. The incidence of hepatoma according to the presence of hepatitis C in CKD-diagnosed patients and whole Sample Cohort

|  |  | | |  | | |
| --- | --- | --- | --- | --- | --- | --- |
|  |  | |  |  | |  |
|  |  |  |  |  |  |  |
|  |  |  |  |  |  |  |
|  |  |  |  |  |  |  |
|  |  |  |  |  |  |  |

**S5 Table**. Comparison of incidence of digestive cancers according to disease definition in data from the National Health Insurance Service-National Sample Cohort with National Cancer Registry data in Korea for 2003

| **Age group** | **Crude rate (per 100,000 person)** | | | **Rate ratio (A/B)** | **Rate ratio (A/C)** | **Rate ratio (B/C)** |
| --- | --- | --- | --- | --- | --- | --- |
|  | **Simple claim† (A)** | **Claim for hospitalization**  **(B)** | **National Cancer Registry (C)** |  |  |  |
| **20-24yrs** | 62.33 | 13.18 | 2.91 | 4.7 | 21.4 | 4.5 |
| **25-29yrs** | 107.74 | 20.04 | 7.84 | 5.4 | 13.7 | 2.6 |
| **30-34yrs** | 131.26 | 29.40 | 18.64 | 4.5 | 7.0 | 1.6 |
| **35-39yrs** | 242.80 | 64.01 | 39.33 | 3.8 | 6.2 | 1.6 |
| **40-44yrs** | 348.26 | 114.31 | 77.07 | 3.0 | 4.5 | 1.5 |
| **45-49yrs** | 515.73 | 179.67 | 135.42 | 2.9 | 3.8 | 1.3 |
| **50-54yrs** | 683.35 | 248.66 | 230.59 | 2.7 | 3.0 | 1.1 |
| **55-59yrs** | 794.40 | 342.37 | 350.59 | 2.3 | 2.3 | 1.0 |
| **60-64yrs** | 959.13 | 484.16 | 487.68 | 2.0 | 2.0 | 1.0 |
| **65-69yrs** | 1180.94 | 648.93 | 641.90 | 1.8 | 1.8 | 1.0 |
| **70-74yrs** | 1292.39 | 793.65 | 775.73 | 1.6 | 1.7 | 1.0 |
| **75-79yrs** | 1470.69 | 920.90 | 822.55 | 1.6 | 1.8 | 1.1 |
| **80-84yrs** | 1319.26 | 824.54 | 845.35 | 1.6 | 1.6 | 1.0 |
| **85+** | 581.18 | 454.05 | 649.47 | 1.3 | 0.9 | 0.7 |
| **Total** | 456.6 | 199.9 | 175.1 | 2.3 | 2.6 | 1.1 |

†A simple claim means that cancer case is first claim with the diagnosis code of digestive cancer, regardless of the inpatient or outpatient.
